# Properties of Macroalgae Biopolymer Films Reinforcement with Polysaccharide Microfibre

**DOI:** 10.3390/polym12112554

**Published:** 2020-10-30

**Authors:** Samsul Rizal, Tze Kiat Lai, Umar Muksin, N.G. Olaiya, C.K. Abdullah, Ikramullah, Esam Bashir Yahya, E.W.N. Chong, H.P.S. Abdul Khalil

**Affiliations:** 1 Department of Mechanical Engineering, Universitas Syiah Kuala, Banda Aceh 23111, Indonesia; ikramullah@mhs.unsyiah.ac.id; 2 School of Industrial Technology, Universiti Sains Malaysia, Penang 11800, Malaysia; tzekiat25@gmail.com (T.K.L.); phunmieoseyemi@gmail.com (N.G.O.); ck_abdullah@usm.my (C.K.A.); essam912013@gmail.com (E.B.Y.); ecarborea929@gmail.com (E.W.N.C.); 3 Department of Physics, Universitas Syiah Kuala, Banda Aceh 23111, Indonesia; muksin.umar@unsyiah.ac.id

**Keywords:** macroalgae, bamboo, hydrophilic, mechanical properties, biodegradable

## Abstract

Developing robust and biodegradable biopolymer films based on macroalgae is a challenging task because of its inadequate mechanical strength and poor moisture barrier attribute to its hydrophilic nature. A promising and sustainable approach to overcome this challenge is to reinforce the biopolymer film with polysaccharide microfibre (microcrystalline cellulose) derived from *Gigantochloa levis* bamboo (GL-MCC). *Eucheuma cottonii* macroalgae were used for the development of biopolymer films without further extraction and purification, which was considered economical and easy. The mechanical, water contact angle (WCA), water absorption capacity (WSC), and thermal behaviour of macroalgae-based biopolymer films revealed that the inclusions of GL-MCC significantly enhanced the durability, moisture barrier, and thermal stability of the biopolymer films. The enhancement is ascribed to the interaction between macroalgae and GL-MCC due to high compatibility. Moreover, the incorporation of GL-MCC successfully increased the rigidity of the macroalgae-based biopolymer films against microorganism and moisture attack, but remain biodegradable and environmental-friendly. The developed biodegradable macroalgae/GL-MCC biopolymer films can potentially be used as packaging materials.

## 1. Introduction

Microcrystalline cellulose (MCC) is a form of cellulose sourced from the plant. Cellulose is the most abundant biopolymer in the world. Cellulose consists of amorphous and crystalline regions. The MCC is the crystalline regions of cellulose. MCC is used in many fields, such as food, biotechnology, pharmaceutical, etc., and is ascribed to its robust mechanical properties, stiffness, non-toxicity, renewability, and biodegradable properties. MCC has been isolated majorly with acid hydrolysis technique due to its shorter reaction time [1,2]. The primary precursor materials for isolation of MCC are cotton and wood. However, these sources are inadequate and limited [1]. At present, bamboo fibres are considered for isolation of MCC because it is renewable, sustainable, and cheap materials.

Bamboo fibres have been reported to significantly enhance the properties and performance of various polymer composites like polypropylene (PP), unsaturated polyester (USP), vinyl ester (VE) resin, polylactic acid (PLA), high-density polyethylene (HDPE), etc. [3]. Literature abounds with evidence reflecting the significance of microcrystalline cellulose (MCC) in properties enhancement of various biopolymer films such as agar [4], starch [5,6], PLA, polybutylene adipate terephthalate (PBAT) [7,8,9], raw seaweed [10], etc. MCC has been established as a new form of cellulose used as reinforcement material in various industrial processes

Malaysia forest has a high diversity of bamboo species. Based on the study of Bahari and Krause [3], Peninsular in Malaysia has about 0.45 million ha or 7% sum of forest area in Malaysia filled with natural bamboo growth. There are about 59 species of bamboo in Malaysia, out of which 25 species are in cultivation, and 34 are indigenous species [9]. Furthermore, only 12 species are commercially utilised species, even though bamboo is one of the most abundant forest resources in Malaysia [3].

*Gigantochloa levis* bamboo, locally known as *Buluh Beting*, is one of the main available bamboo species in Malaysia. This bamboo can grow up to 20 m tall with a diameter up to 16 cm or even more. *Gigantochloa levis* is among the best bamboo candidates for cultivation and exploitation since it can produce strong and straight culm bamboo shoot. Traditionally, *Gigantochloa levis* bamboo culm had been used to produce many products, such as rafts, fish trap, baskets, musical instruments, handicrafts, and furniture [3]. Nevertheless, no MCC extraction study has been done on this bamboo species up to date. Owing to its abundance in Malaysia’s forest, this bamboo is considered readily available and cheap raw materials for various industries, especially to produce microcrystalline cellulose (MCC). The raw macroalgae is renewable, sustainable, and economically viable due to high yield precursor plant. This makes this production process cheap [11].

Synthetic plastics used for packaging in foods and goods are non-biodegradable [12,13]. It has been reported as the cause of significant environmental pollution worldwide. Despite its high demand, synthetic plastic waste recycling is not cost-effective and requires complex manufacturing procedures [14,15]. As a result, this has led to the interest in green, renewable, and sustainable macroalgae biopolymers that can help to reduce the plastic waste and control the carbon dioxide emission [5,16].

Currently, macroalgae derivatives like agar, carrageenan, and alginate have been proposed for packaging materials application. Many researchers had reported the potential usage of macroalgae derivatives for biopolymer films development [4,17,18,19]. Yet, the use of macroalgae biopolymer for biodegradable films application has been restricted due to their low mechanical durability, poor water barrier, long and pricey extraction, and purification process [20]. However, raw Eucheuma *cottonii* without extraction and purification was used for biopolymer film development in this study. the use of raw Eucheuma *cottonii* is cost-effective, ease processability, and environmental-friendly.

Furthermore, this work develops renewable and biodegradable macroalgae-based films with enhanced mechanical, thermal, and water barrier properties using bamboo MCC as reinforcement fillers. The uniqueness of this study is in the use of raw macroalgae, instead of its derivatives, for biopolymer films fabrication which is economical and removes complex processing. The effects of the GL-MCC in raw macroalgae biopolymer films have not been reported. The properties of the developed film were analysed with different concentration of the loaded filler in the raw macroalgae matrix.

## 2. Materials and Methods

Raw macroalgae *Eucheuma cottonii* was purchased from Tawau, Sabah (East Malaysia). Matured *Gigantochloa levis* bamboo used in the isolation of polysaccharide microfibre (GL-MCC) was obtained from local suppliers at Taman Melawati Area (Kuala Lumpur, Selangor, Malaysia). Polysaccharide microfibre (GL-MCC) was prepared through a combination of pulping, bleaching, and acid hydrolysis process at the laboratory of School of Industrial Technology, Universiti Sains Malaysia (USM). All the following chemicals and solvents used in this study were of analytical grade (AR); sodium hydroxide pellet (NaOH), sodium chlorite flakes (NaClO_2_), acetic acid (CH_3_ COOH, 98%), anthraquinone (AQ) hydrochloric acid (HCl) (37%), and glycerol (99.9%, Sigma Aldrich, MO, USA).

### 2.1. Isolation of Gigantochloa Levis Bamboo-MCC (GL-MCC)

Hermawan et al.’s [10] method was used to isolate MCC from *Gigantochloa levis* bamboo. Bamboo was air-dried for 7 days until constant weight to remove excess moisture before undergo pulping. Bamboo (300 g) were pulp with 23% of NaOH and 0.1% of anthraquinone at a temperature of 160 °C for 2 h. Then, brownish bamboo pulps were bleached using sodium chloride (3 g) and acetic acid (5%) at 85 °C for another 2 h, according to Suvachittanont and Ratanapan’s [21] method. Pachuau et al.’s [22] method was used to isolate α-cellulose from bamboo. Finally, the α-cellulose was subjected to acid hydrolysis following the Chuayjuljit et al. [23] approach.

### 2.2. Preparation of Macroalgae (SW)/Polysaccharide Microfibre (GL-MCC) Biopolymer Film

Four grams (4 g) of oven-dried raw macroalgae was dissolved in 200 mL of distilled water with the addition of 2 g of glycerol and then heated at 85 °C with constant stirring till the suspension congealed. Glycerol provides the plasticiser effect to the macroalgae (SW)/*Gigantochloa levis*-MCC biopolymer film. The concentration of fillers used in the macroalgae biopolymer films were 2%, 4%, 6%, 8%. After, the hot solution was cast on the plate and oven-dried at 45 °C for 24 h. The dry macroalgae biopolymer films were peeled off from the plate and conditioned in a desiccator at 50% relative humidity for 48 h. Test samples were cut into standard sizes and analysed.

### 2.3. Moisture Content (MC)

Pachuau et al.’s [22] reported process was used to analyse the MC of the GL-MCC powder. Initially, 2 g of GL-MCC powder were weighed and dried in an oven at 105 °C for 5 h to remove excess moisture. The GL-MCC powder was analysed in triplicate. Macroalgae/GL-MCC biopolymer film MC was also determined by measuring the weight loss of the biopolymer films before and after drying in an oven at 105 °C until a constant weight was achieved. Samples were analysed in triplicate, and the results presented in percentage (%) by weight.

### 2.4. Water Absorption Capacity (WSC)

Ghanbarzadeh and Almasi’s [24] method was used to determine the WSC of the macroalgae (SW)/polysaccharide microfibre (GL-MCC) film. The moisture loss (S_i_) of the macroalgae (SW)/polysaccharide microfibre (GL-MCC) film was obtained from oven-dried samples at 105 °C until constant weight. After weighing, the dried samples were conditioned in a desiccator with distilled water for 48 h at room temperature. Following this, the samples were reweighed (S_f_). The WSC of the biopolymer films was calculated with Equation (1).

(1) $$\mathrm{WSC}\;\left(\%\right)=\left(\frac{S_{f}-S_{i}}{S_{i}}\right)\times 100$$

### 2.5. Film Thickness

A manual digital micrometre (0.001 mm) was used to measure the thickness of the macroalgae biopolymer films. The results of the averaged sample thickness were recorded as mean (µm) ± standard deviation (SD).

### 2.6. Morphological Property

The morphological property of the macroalgae (SW)/polysaccharide microfibre (GL-MCC) biopolymer film was determined with a scanning electron microscope (SEM). Before observing the surface morphology, the film sample was cut into 10 mm × 10 mm and oven-dried at 60 °C. The morphological property of polysaccharide microfibre (GI-MCC) was also determined using SEM, and their length sizes were measured using Image J software (LOCI, J1.5k, Madison, WI, USA). The atomic force microscope (AFM) was used to measure the surface roughness (R_q_) of the films. The film samples (3 × 3 cm^2^) were placed on freshly cleaved mica and analysed at a 0.3 Hz scan rate. The three-dimensional (3D) images from the analysis were obtained at 12.3 (nm) and scan area of 30 µm.

### 2.7. X-Ray Diffraction Micrographs (X-RD)

The crystallinity of the GL-MCC and macroalgae (SW)/ polysaccharide microfibre (GL-MCC) biopolymer films were determined with X-Ray Diffraction micrographs (X-RD). The GL-MCC powder and macroalgae/GL-MCC biopolymer films were scanned at room temperature and a diffraction angle range of 2θ = 10° to 50°.

### 2.8. Thermogravimetric Analysis

The thermal properties of GL-MCC and the macroalgae (SW)/polysaccharide microfibre (GL-MCC) biopolymer film were measured with the thermogravimetric analyser (TGA). A sample of 10 mg by weight was measured and put in a standard cup. This was placed in the thermogravimetry analyser with a pre-weighed empty cup as a reference, and the samples were heated from 30 °C to 800 °C. A heating rate of 10 °C/min was used, and the weight loss and derivative weight loss with temperature was obtained under nitrogen.

### 2.9. Tensile Properties

The tensile strength (TS), tensile modulus (TM), and elongation at break (EAB) of the macroalgae (SW)/polysaccharide microfibre (GL-MCC) biopolymer films were measured based on the ASTM D882-02. The macroalgae biopolymer films were chopped into strips of 1 cm width × 15 cm length and were conditioned at 50% relative humidity (RH) for a week before being analysed. An initial grip distance of 100 mm and a test speed of 100 mm/min were applied for the test. Five replicated measurements were recorded per sample, and the average calculated.

### 2.10. Water Contact Angle (WCA)

The WCA of the macroalgae (SW)/polysaccharide microfibre (GL-MCC) biopolymer film was determined by measuring the water contact angle (WCA) of the film surface using a CA analyser at room temperature. The contact angle is an inverse measurement of wettability. Each macroalgae/polysaccharide microfibre (GL-MCC) biopolymer film was cut into square pieces (3 × 3 cm^2^) and placed on the flat stage attached to the CA analyser. Measurement was taken in triplicate.

### 2.11. Soil Burial Test of Macroalgae (SW)/Polysaccharide Microfibre (GL-MCC) Biopolymer Films

**Figure 1:**
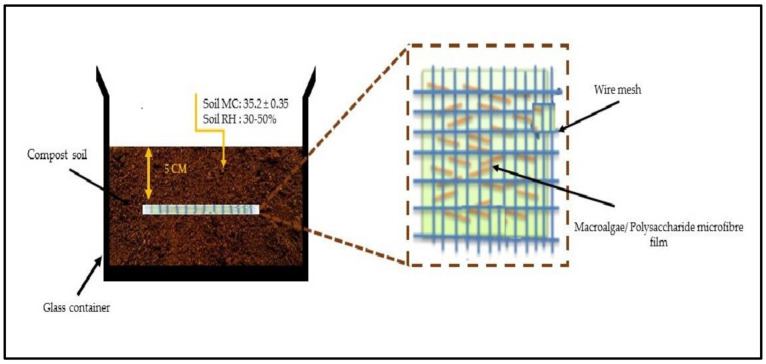
Illustrative diagram soil burial test of macroalgae (SW)/polysaccharide microfibre (GL-MCC) films.

A soil burial test was carried out on a laboratory scale for macroalgae (SW)/polysaccharide microfibre (GL-MCC) biopolymer film based on the method of Tan et al. [25] with some modification. Dark garden soil of 35 °C (*w*/*w*) moisture content was poured into a 1000 mL plastic container, as shown in Figure 1. The dark garden soil composed of topsoil, rich humus, cocopeat, charcoal powder, and river sand, which is suitable for planting fruits and vegetables. Following this, all film samples (3 × 3 cm^2^) were weighed (W_0_) before buried in the soil at a depth of 5 cm from the surface. At different time intervals (7, 14, 21, and 30 days), the biopolymer film was dug out from the container and washed [26,27]. The samples were dried for 24 h at 50 °C and kept in a desiccator to cool before being weighed. The percentage weight loss of the macroalgae biopolymer film was obtained with Equation (2), where W_0_ indicates the initial weight of the sample before the soil-burial test and W_1_,is the weight of the sample residue after the testing.

(2) $$Weight\;loss\;\left(\%\right)=\left(\frac{W_{0}-W_{1}}{W_{1}}\right)\times 100$$

### 2.12. Fourier Transforms Infrared Spectroscopy (FT-IR) Analysis

The functional group of samples (GL-MCC and SW/GL-MCC biopolymer films) were analysed with Fourier Transforms Infrared Spectrophotometer (PerkinElmer, PC1600, Winter Street Waltham, MA, USA). The transmittance spectrum of FT-IR analysis samples was prepared with one milligram (mg) of GL-MCC, mixed with potassium bromide (KBr), pressed into flat film, and then analysed. SW/GL-MCC biopolymer films were also cut into 3 × 3 cm^2^ sizes and oven-dried at 60 °C for 24 h before the FT-IR analysis. All samples were analysed between a range of 400–4000 cm^−1^.

### 2.13. Statistical Analysis

The results of this study were statistically analysed using significant one-way variance (ANOVA). The significance difference (*p* ≤ 0.05) of each mean value was determined with Tukey’s HSD multiple comparisons test using the IBM SPSS statistics Data Editors.

## 3. Results and Discussion

### 3.1. Moisture Content (MC)

The moisture content (MC) of the GL-MCC was 4.65%. It was almost similar compared to those obtained from Muli (*Melocanna baccifera*) and Rawnal (*Dendrocalamus longispathus*) bamboo MCC (4.22 to 4.87%) reported by Pachuau et al. [20], yet it was much lower than other fibres’ of MCC, such as raw cotton (*Cochlospermum planchoii*) (7.2%) and bark of the water gourd *Lageriana siceraria*- MCC (9.0%) [28,29]. This observation can be majorly attributed to the difference in properties of raw materials. The low MC of GL-MCC was desirable for tablets forming in medicine because high MC could jeopardise the mechanical properties of MCC, which eventually would reduce the strength of the tablets formed [30]. Moreover, the presence of excess moisture in the MCC was discouraged because it could trigger fungal growth and resulted in deterioration of the samples.

**Figure 2:**
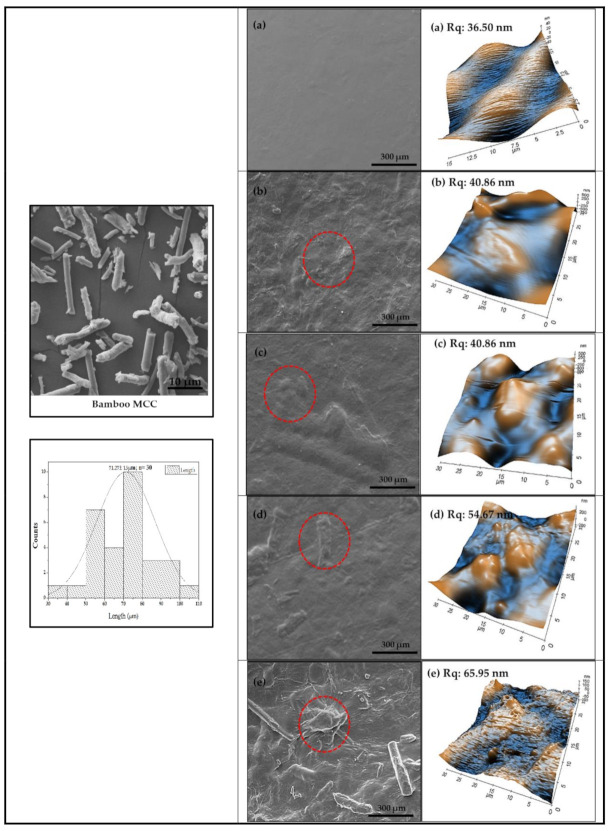
The surface morphologies of macroalgae (SW)/polysaccharide microfibre (GL-MCC) biopolymer films by scanning electron microscope (SEM) and atomic force microscope (AFM) for (**a**) Plain-SW, (**b**) SW/2% GL-MCC, (**c**) SW/4% GL-MCC, (**d**) SW/6% GL-MCC, and (**e**) SW/8% GL-MCC biopolymer films.

The MC of macroalgae (SW)/polysaccharide microfibre (GL-MCC) biopolymer films were shown in Table 1. The MC of the plain macroalgae (plain-SW) biopolymer film was 38.17 ± 0.6%, and it decreased significantly (*p* < 0.05) when GL-MCC was incorporated. The lowest MC was archived at SW/4% GL-MCC biopolymer film. The strong intermolecular hydrogen bonding between the hydroxyl group of GL-MCC and the macroalgae (SW) matrix had reduced the free available hydroxy group. This decreased the MC of the SW composite films. Moreover, GL-MCC is usually less hydrophilic with low MC ascribe to its high crystalline property. This finding is consistent with those reported by Shankar and Rhim [4]. Reddy and Rhim [31] also found that the MC of agar film was significantly improved due to the inclusions of cellulose nanocrystal (CNC) up to 5% CNC filler loading. In this study, the reversed MC results were found at SW/6% GL-MCC and SW/8% GL-MCC biopolymer film. The agglomeration of GL-MCC in SW/6% GL-MCC and SW/8% GL-MCC biopolymer film, as shown by Figure 2 in SEM analysis, probably had weakened the GL-MCC(filler)-SW(matrix) interaction, and caused exposure of a more free available hydroxyl group (–OH). Consequently, it increased the MC of the SW/GL-MCC biopolymer films.

The thickness (µm) of the plain-SW film was 76 ± 0.003 µm. Macroalgae (SW)/8% GL-MCC biopolymer films have the highest thickness of 108 ± 0.006 µm. The thickness of the macroalgae biopolymer film was gradually increased with the increase in fillers loading. This finding is compatible with those reported by Shankar and Rhim [16] following by incorporating of nanocellulose (NC) and microcrystalline cellulose (MCC) in agar-based films. Hermawan et al. [10] suggested that large structure properties of MCC fillers might enlarge the interstitial spacing between the macroalgae (SW) polymer chains and further create a less close-packed structure, which eventually increased the thickness of the macroalgae/polysaccharide microfibre (GL-MCC) biopolymer films. In this study, the SW/GL-MCC biopolymer film did not exhibit significant (*p* > 0.05) film increment, except for SW/8% GL-MCC biopolymer films, which was about 32 µm thicker than their plain-SW biopolymer films.

### 3.2. Water Sorption Capacity (WSC)

The WSC of GL-MCC was 9.00% for the first day of exposure and tended to increase up to 12.50% on day 5. This value was lower compared to agriculture waste of Mexican sunflower *Tithonia diversifolia* (14.60%), banana stem *Musa sapientum* (14.31%), and *Musa paradisiaca* (13.91%) and commercial Avicel PH 101 (16.6 to 22.80%) [29]. Although GL-MCC was highly crystalline, it was a water-sensitive material. The moisture sensitivity of the material was estimated by their water absorption capacity (WSC). The low WSC of GL-MCC indicates that it is highly crystalline with low amorphous cellulose content because the crystalline phase of the cellulose usually does not absorb as much water as the amorphous phase. This lower water sensitivity of GL-MCC could probably enhance the tensile and moisture barrier of the macroalgae biocomposites film afterwards.

Like most polysaccharide films, the macroalgae-based film was water-sensitive with a high WSC capacity of 180% due to its hydrophilic nature. The incorporation of GL-MCC significantly (*p* < 0.05) minimised the WSC of the macroalgae biopolymer films. The most pronounced value was found at macroalgae (SW)/4% GL-MCC biopolymer films whereby the WSC of the film dropped to 144%, with a total of 44% reduction followed by the incorporation of GL-MCC. Despite the macroalgae (SW)/6% GL-MCC and macroalgae (SW)/8%, GL-MCC biopolymer films showed a slight increase of WSC after a threshold level. Still, it was lower compared to their plain-SW. This finding was in agreement with those reported by Wilpiszewska and Czech [6] whose confirmed that the decrease of WSC of the starch-based films due to reinforcement with MCC.

### 3.3. Morphological Properties of Polysaccharide Microfibre (GL-MCC) and Macroalgae (SW)/Polysaccharide Microfibre (GL-MCC) Biopolymer Films

The morphological property of GL-MCC was displayed in Figure 2. The GL-MCC showed an irregular, elongated shape with a rough surface. Figure 2 shows the histogram of GL-MCC length counts, using Image J software. The graph shows that most of the GL-MCC ranged from 50 µm to 80 µm, with a mean value of 71.27 ± 15 µm. GL-MCC tends to exhibit fibrous and rod-shaped morphological properties similar to *Muli* and *Rawnal* bamboo MCC reported by Pachuau et al. [22]. Shankar and Rhim [4] also reported the irregular shape of microcrystalline cellulose (MCC) with the size in the range of 20 to 200 µm.

Morphology of macroalgae (SW)/polysaccharide microfibre (GL-MCC) biopolymer films was observed using SEM, and the results were displayed in Figure 2. Plain-SW films exhibited even a homogenous and compact surface without any cracks or voids. Following the incorporation of GL-MCC fillers, the surface of the macroalgae (SW) biopolymer films became rougher and uneven. GL-MCC was embedded compactly in the SW matrix. As the filler loading increased, the surface of the SW/GL-MCC biopolymer films became more irregular, and the aggregations of GL-MCC was observed at SW/8% GL-MCC biopolymer films. This finding was in agreement with morphological properties of agar/ MCC and agar/CNC biocomposites films reported by Shankar and Rhim [4] and Reddy and Rhim [31]. The topography of the macroalgae/GL-MCC composite films was determined using AFM. The plain-SW films exhibit a smooth surface with surface roughness value (R_q_) of 36.50 nm. The SW/8% GL-MCC biopolymer films having the highest R_q_ value of 65.95 nm might attribute to the incorporation of high concentration large GL-MCC filler. Moreover, the agglomeration of GL-MCC filler in the SW matrix could also contribute to the high R_q_ values. The results obtained reflected the changes in macroalgae structure due to aggregation formation by GL-MCC and its loading properties.

### 3.4. The Crystallinity Index of GL-MCC and SW/GL-MCC Biopolymer Films

**Figure 3:**
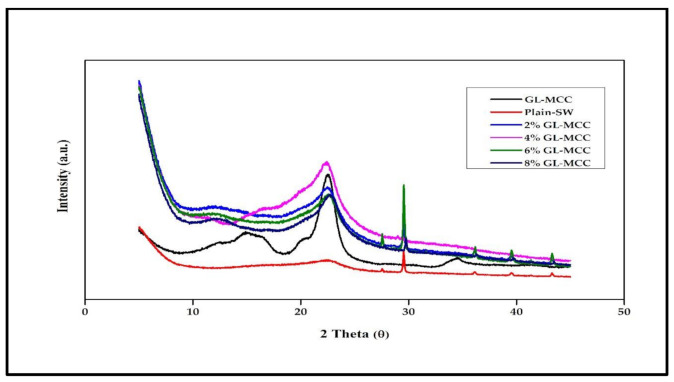
X-RD patterns of macroalgae (GL-MCC) and macroalgae (SW)/polysaccharide microfibre (GL-MCC) biopolymer films.

The crystallinity index of GL-MCC was 76.84 ± 1.1%. The enhanced GL-MCC crystallinity index reflects the successful elimination of its amorphous part by acid hydrolysis process. The crystallinity index of GL-MCC was slightly higher than Muli bamboo-MCC (68%) and Rawnal bamboo-MCC (72.5%), as reported by Pachuau et al. [22]. The different crystallinity index showed by different bamboo species is attributed to the different MCC isolation method. From Figure 3, the major crystalline peak of GL-MCC occurred at 2θ = 20° to 25°. This peak reflects the cellulose I structure of GL-MCC and designate the purity of the GL-MCC has been preserved [32].

The plain-SW biopolymer film exhibited broadband at 2θ = 20° to 25°, represents the amorphous nature of macroalgae. Other peaks around 2θ = 29°, 35°, and 42° presence in plain-SW spectra were mostly due to the presence of minerals like potassium chloride and sodium chlorite in macroalgae itself [33]. Due to the existence of calcite, a very sharp peak was found at 2θ = 28° to 30° in plain-SW spectra. Calcite has been reported as a calcium carbonate mineral salts present in macroalgae [34].

**Figure 4:**
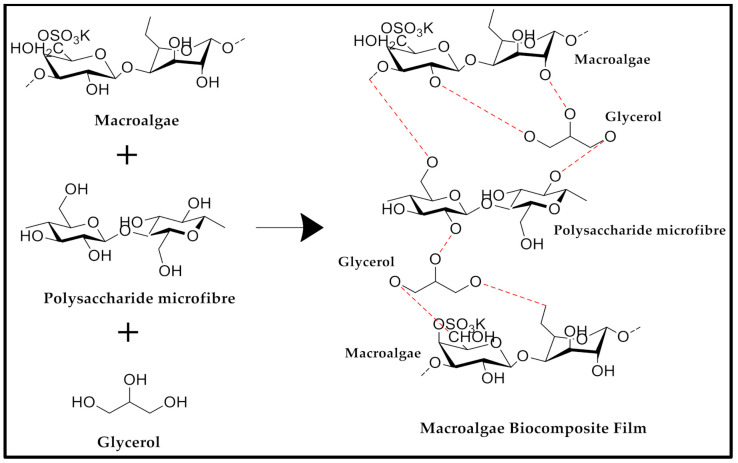
Schematic representation of intermolecular interaction between macroalgae (SW) and polysaccharide microfibre (GL-MCC) in the presence of glycerol.

Initially, the plain-SW spectra showed a less prominent peak at 2θ = 20° to 22.5°. Due to the incorporation of GL-MCC, the peak intensity (2θ = 20° to 22.5°) became more prominent. The sharp peak was observed at 2θ = 20° to 22.5° for 2% to 8% SW/GL-MCC composite films. These results reflected that the GL-MCC had enhanced the relative crystallinity of the plain-SW films. This finding was consistent with Santos et al. [35]. It was reported by Santos et al. [35] that the peak intensity at 2θ = 22.6° for polylactic acid (PLA) films showed a gradual increase due to inclusions of MCC and CNC fillers. This GL-MCC might act as a nucleating agent for crystallisation of plain-SW composite films since the plain-SW films without any crystalline cellulose reinforcement presented amorphous behaviour. Moreover, the robust hydrogen bonding formed by high crystallinity GL-MCC and amorphous macroalgae matrix, as depicted in Figure 4, could eventually enhance the relative crystallinity of the SW/GL-MCC biopolymer film.

### 3.5. Thermal Properties of GL-MCC and Macroalgae (SW)/GL-MCC Biopolymer Films

**Figure 5:**
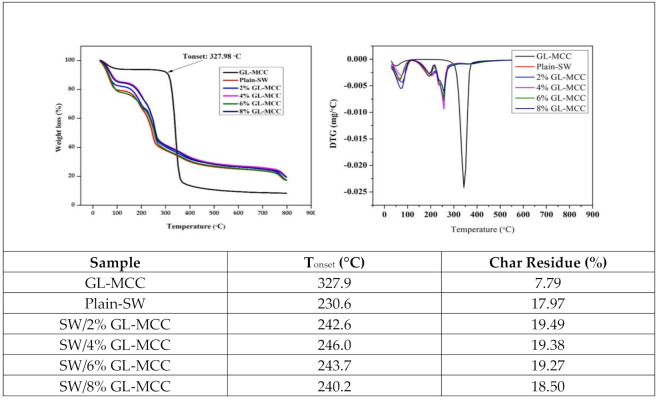
Thermal properties of GL-MCC and macroalgae (SW)/ polysaccharide microfibre (GL-MCC) biopolymer films.

The results of the thermogravimetry analysis (TGA) and derivative thermogravimetry (DTG) of GL-MCC and SW/GL-MCC biopolymer films are shown in Figure 5. The thermal degradation of the GL-MCC showed two steps. The initial stage of degradation occurred at around 30 °C to 100 °C. This was ascribed to the elimination of moisture trapped within the samples [4,31]. The main degradation stage has occurred in the range of 300 °C to 400 °C. In this study, the T_onset_ for GL-MCC was 327.98 °C and its T_max_ was 336.80 °C.

The weight loss of about 89.88% was obtained at this major degradation stage. This was attributed to the thermal decomposition of cellulosic materials [4]. No weight loss (%) peak observed at 450 °C and above, which related to the degradation of lignin content, indicating that GL-MCC produce in this study was free from lignin. High T_onset_ and T_max_ signify the high thermal stability of a material. Thus, GL-MCC was more thermally stable compared to Muli bamboo-MCC (315.74 °C) reported by Pachuau et al. [22], and this was attributed to the high crystallinity of GL-MCC as shown in the X-RD result.

All SW/GL-MCC biopolymer films exhibited four stages of thermal degradation, as shown by TGA and DTG curves in Figure 5. The initial phase of degradation occurs at 50 °C to 65 °C, which represented about 10–20% of weight loss. At this stage, moisture was removed from the macroalgae films. This finding was further confirmed by Shankar and Rhim [4] and Zarina and Ahmad [17] on agar/MCC biopolymer films and carrageenan/CNC biopolymer films. The weight reduction of about 15% to 30% at 160 °C to 202 °C was attributed to the degradation of glycerol [36]. The third decomposition occurred at 225 °C to 257 °C and was ascribed to the degradation of macroalgae polysaccharides [17]. Based on Figure 5, the thermal degradation of plain-SW films was 230.6 °C increased to 246 °C, followed by incorporation of 4% GL-MCC. The T_max_ (DTG) for SW/GL-MCC biopolymer films were in range of 245 °C to 252 °C, which was higher than the plain-SW. With the incorporation of GL-MCC, the onset degradation temperature (T_onset_) and maximum degradation temperature (T_max_) were shifted to a higher temperature with the increased percentage of GL-MCC. This showed that the thermal stability of the plain-SW films was enhanced with the incorporation of GL-MCC. The enhanced thermal stability is probably due to the strong intermolecular interactions between macroalgae matrix and GL-MCC. The bond formed increased the energy required to break the intermolecular bonding for degradation of the composite, and thereby led to higher T_onset_ and T_max_. A similar finding was reported by Zarina and Ahmad [17] with the incorporation of cellulose nanocrystals (CNCs) into carrageenan-based films.

### 3.6. Tensile Properties of Macroalgae (SW)/GL-MCC Biopolymer Films

**Figure 6:**
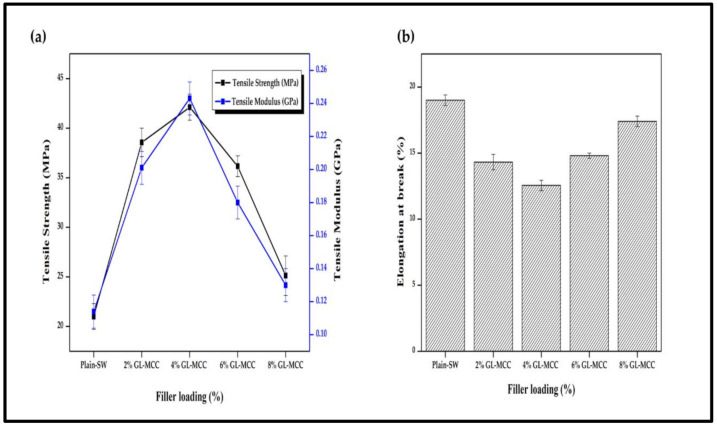
(**a**) Tensile strength and tensile modulus; (**b**) elongation at break of macroalgae (SW)/polysaccharide microfibre (GL-MCC) biopolymer films.

The tensile properties of the macroalgae (SW)/polysaccharide microfibre (GL-MCC) biopolymer films were shown in Figure 6a,b. The tensile strength (TS) and tensile modulus (TM) of plain macroalgae were 21.00 MPa and 0.114 GPa, respectively, tend to increase up to the optimum level of 42.12 MPa and 0.243 GPa, respectively due to incorporation of 4% of GL-MCC. The TS and TM of SW/GL-MCC biopolymer film were decreased when the filler loading increased to 6% and 8%. The TS and TM of SW/4% GL-MCC biopolymer films were significantly higher (*p* < 0.05) compared with their plain macroalgae films, which could be explained by good dispersion of GL-MCC in macroalgae matrix as revealed by SEM analysis. Moreover, excellent compatibility between GL-MCC and macroalgae matrix due to their similar chemical properties also enhances the tensile strength of the biopolymer films.

Additionally, robust intermolecular hydrogen bonding formation of hydroxyl group GL-MCC and macroalgae matrix, as depicted in Figure 4, might improve the tensile properties of the SW biopolymer films as well. Furthermore, the reduction of elongation at break (EAB) of SW/GL-MCC biopolymer films confirmed that the strong filler-matrix interaction had restricted the mobility of the SW polymer strand and eventually caused the stiffness (tensile modulus) of the SW/GL-MCC biopolymer films to increase. Based on Figure 6b, the EAB of plain macroalgae film was reduced significantly (*p* < 0.05) due to the incorporation of 4% GL-MCC. However, at high GL-MCC loadings (6% and 8%) the EAB of macroalgae biopolymer film was slightly increased. This phenomenon could be due to the agglomeration of GL-MCC in the macroalgae matrix at a high concentration that diminishes the strong filler-matrix interaction. Zarina and Ahmad [17] also reported similar tensile properties for the carrageenan/CNCs composite films due to the incorporation of cellulose nanocrystals (CNCs). According to Zarina and Ahmad [17], TS and TM of kappa-carrageenan films increase during their EAB decrease due to the incorporation of CNCs. They revealed that 4% was their optimum filler loading, and above the threshold level; the TS, TM, and EAB showed reverse results.

### 3.7. Wettability of Macroalgae (SW)/Polysaccharide Microfibre (GL-MCC) Biopolymer Films

The water contact angle (WCA) is the inverse measure of the wettability of polymer films. The water contact angle of plain macroalgae films and SW/GL-MCC biopolymer films was measured with contact angle analyser, and the results were shown in Table 2. The WCA of plain macroalgae film was 42.00 ± 0.53° and was significantly increased (*p* < 0.05) to 60.80 ± 0.05° with the incorporation of 4% GL-MCC. The further inclusion of GL-MCC caused a slight reduction of WCA. The WCA of SW/6%GL-MCC and SW/8%GL-MCC biopolymer films was 54.76 ± 0.50° and 50.12 ± 0.03 °C, respectively. The enhancement of WCA followed by incorporation of GL-MCC might due to the formation of the robust intermolecular hydrogen bonding of GL-MCC and macroalgae hydroxyl group (–OH).

This type of formation caused a reduction of the free available hydroxyl group in macroalgae to attach to the surrounding water molecules. Therefore, it can efficiently decrease the hydrophilicity of the macroalgae biopolymer films. This finding was consistent with those reported by Shankar and Rhim [4] on agar/NC composite films. The material having WCA more than 90° is considered hydrophobic. The macroalgae/GL-MCC biopolymer films were categorised as hydrophilic material with moderate wettability because of having WCA less than 90°.

### 3.8. Biodegradability of Macroalgae (SW)/Polysaccharide Microfibre (GL-MCC) Biopolymer Films

**Figure 7:**
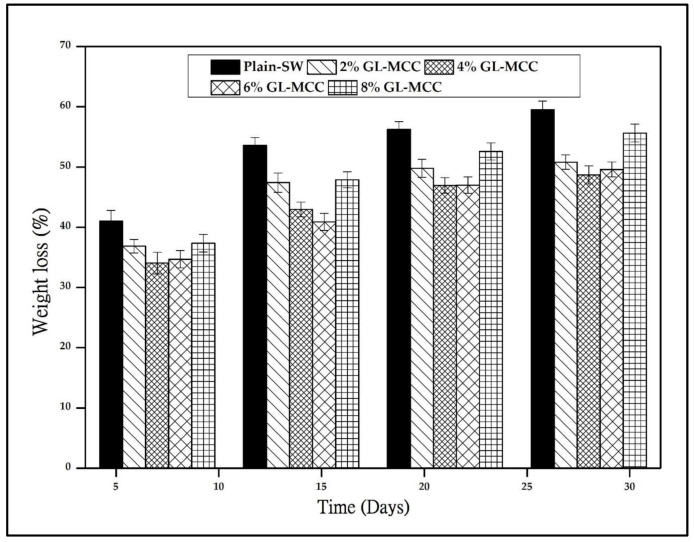
The mass reduction (%) of macroalgae (SW)/polysaccharide microfibre (GL-MCC) biopolymer films.

The weight reduction (%) of macroalgae (SW)/GL-MCC biopolymer films at a different time interval of 7, 14, 21, and 30 days were shown in Figure 7. The plain macroalgae (SW) biopolymer films showed 41% weight reduction at day seven and continue to increase up to 59.54% at day 30. The hydrophilic nature and amorphous property of plain SW biopolymer films triggered the biodegradable property of the biopolymer films. However, the SW/GL-MCC biopolymer films showed lower film mass reduction compared to their plain-SW composite films. The mass reduction for SW/4% GL-MCC biopolymer films for day 7, 14, 21, and 30 was 34.05%, 42.96%, 46.95%, and 48.67%, respectively. This phenomenon was due to robust interfacial adhesion of GL-MCC filler- macroalgae matrix attributed to their high compatibility.

Based on the X-RD analysis, the plain-SW biopolymer films composed mainly of the amorphous phase. The incorporation of GL-MCC tends to increase the crystallinity of the films. The strong filler-matrix interaction strengthens the strength and rigidity of the SW films. As a result, the microorganism might find difficulties or take a long time to disintegrate those crystalline phases in macroalgae/GL-MCC biopolymer films. Hence, the mass reduction (%) of macroalgae/GL-MCC biopolymer films were less favourable compared to their plain macroalgae biopolymer films. This finding was consistent with those reported in the work of Deepa et al. [37]. In the report of Deepa et al. [37], the rate of weight loss decreases in alginate films due to the incorporation of cellulose nanofibrils (CNF). They reported that the interaction between CNF fillers and alginate matrix was the main reason for the lower weight loss.

Despite that, the WSC of plain macroalgae biopolymer films was very high. However, they tend to reduce due to the incorporation of GL-MCC at 4% concentration. The composite that is susceptible to absorb moisture like plain macroalgae (plain-SW) films is more susceptible to weight loss because it can stimulate microorganism growth. However, the incorporation of GL-MCC had diminished the WSC of the macroalgae-based films to some extent. This eventually hindered the accessibility of microorganisms and consequently slowed down the mass reduction of SW/GL-MCC biopolymer films, although the GL-MCC is seen to lower down the mass reduction of SW biopolymer film. Yet, it did not hinder the biodegradation property of macroalgae (SW)/GL-MCC biopolymer films.

**Figure 8:**
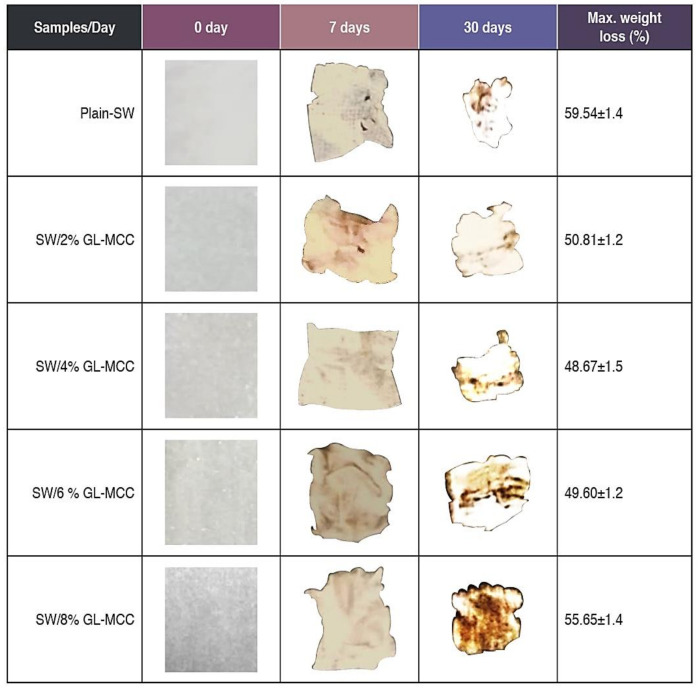
Changes of the appearance of SW/GL-MCC biopolymer films before and after soil-burial testing.

The digital photos of plain macroalgae and SW/GL-MCC biopolymer films were presented in Figure 8. Initially, all samples have clear, smooth, and white surfaces. Rapid shrink and cracks happen to the samples after 7 days of the soil-burial test. The colour tone and surface structure of the SW/GL-MCC biopolymer films were changed. More ruptures and cavities started to form, especially at the edges of the sample, as shown in Figure 8. The samples were turned from white and clear into brownish with a noticeable reduction of sample sizes after 30 days of testing. The macroalgae (SW)/GL-MCC undergo rapid deterioration and deformation comparable to their plain macroalgae films.

### 3.9. FT-IR Characterisation of Macroalgae (SW)/Polysaccharide Microfibre (GL-MCC) Biopolymer Films

**Figure 9:**
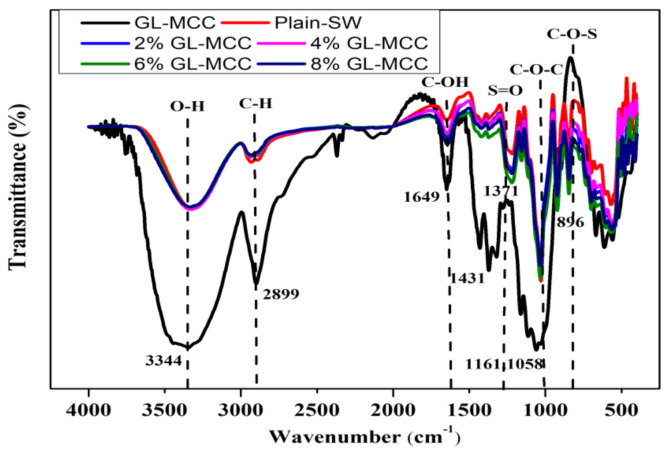
Fourier Transforms Infrared Spectroscopy (FT-IR) characterisation of macroalgae (SW)/polysaccharide microfibre (GL-MCC) biopolymer films.

Figure 9 depicts the IR spectra of GL-MCC and the SW/GL-MCC biopolymer films. The strong broadband was observed at 3500 cm^−1^ for all samples, which is assigned to different O–H stretching modes. Another strong peak around 2900 cm^−1^ is attributed to the C–H stretching vibrations. This asymmetric and symmetric methyl and methylene stretching groups could be found at around 2899 to 2900 cm^−1^ for both GL-MCC and SW/GL-MCC biopolymer films. The O–H stretching vibration and C–H rocking vibration shown in the spectra of GL-MCC represented the typical characteristic structure of cellulose [36]. In this study, the peak for plain-SW biopolymer films exhibited wavenumber of 3323 cm^−1^ tends to shift to a higher wavenumber, which is 3331 cm^−1^ corresponding to the GL-MCC filler loaded. This was probably due to the formation of hydrogen bonding between –OH groups from the macroalgae and GL-MCC filler within the matrices. Huq et al. [14] reported similar functional groups in their study on the properties of integrated crystalline cellulose (NCC) in alginate film matrix.

Moreover, all SW/GL-MCC biopolymer films exhibited peaks at 1217–1219 cm^−1^ which shows the presence of sulphate (S=O) from the kappa-carrageenan present in macroalgae [12]. The peak at 1371 cm^−1^ in the GL-MCC filler could also probably be related to the –CH or C–O bonding vibrations in the polysaccharide’s aromatic rings [20]. Furthermore, for GL-MCC, another peak at 896 cm^−1^ shows the presence of C–H out of plane stretching of cellulose [20]. The appearance of this peak indicates that the cellulose molecules were not chemically modified during the isolation of MCC from GL bamboo. The sharp peaks between 1031 cm^−1^ and 844 cm^−1^ could be due to the glycosidic linkage (C–O) of 3, 6-anhydrous-D-galactose, C–O–S stretching in a (1–3)-D-galactose and C–O–C stretching in 3,6-anhydrogalactose. These peaks are typically indicators of the presence of carrageenan in macroalgae biopolymer films [12]. Overall, the plain-SW and SW/GL-MCC biopolymer film spectra showed a relatively similar pattern of bands, and this is an indication of good miscibility between the GL-MCC and macroalgae polymer matrix.

## 4. Conclusions

The polysaccharide microfibre (GL-MCC) was derived from *Gigantochloa levis* bamboo. The physicochemical, thermal, and morphological properties of polysaccharide microfibre (GL-MCC) were successfully characterised by X-RD, TGA, FT-IR, and SEM. Raw macroalgae can use for the development of biopolymer films. The reinforcement effect of different filler loading in macroalgae biopolymer films was confirmed. The polysaccharide microfibre (GL-MCC) significantly enhanced the mechanical, water barrier, and thermal properties of the macroalgae biopolymer films. The 4% was the optimum filler loading for polysaccharide microfibre (GL-MCC) in macroalgae composite films. The improved macroalgae (SW)/polysaccharide microfibre (GL-MCC) biopolymer films can potentially use for packaging material applications.

## Figures and Tables

**Table 1:** Moisture content, water sorption capacity, and thickness of macroalgae/GL-MCC biopolymer films.

Film	MC (%)	WSC (%)	Thickness (µm)
Plain-SW	38.17 ± 0.6 ^d^	180 ± 3.0 ^b^	76 ± 0.003 ^a^
SW/2% GL-MCC	31.43 ± 1.8 ^c^	140 ± 4.0 ^a^	93 ± 0.004 ^a b^
SW/4% GL-MCC	23.83 ± 1.6 ^b^	136 ± 3.0 ^a^	102 ± 0.003 ^ab^
SW/6% GL-MCC	25.30 ± 0.9 ^a^	146 ± 3.0 ^a^	105 ± 0.005 ^ab^
SW/8% GL-MCC	27.50 ± 0.2 ^a^	158 ± 5.0 ^a^	108 ± 0.006 ^b^

Note: Any mean with different superscript letter a, ab, b, c, and d indicates significant difference (*p* < 0.05).

**Table 2:** Water contact angle (°) of macroalgae (SW)/polysaccharide microfibre (GL-MCC) biopolymer films.

Samples	Droplet Image	Contact Angle (°)	CA ± SD
Plain-SW	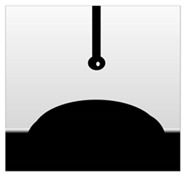	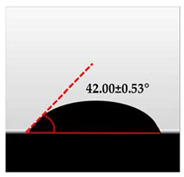	42.00 ± 0.53 ^a^
SW/2% GL-MCC	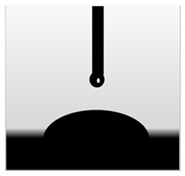	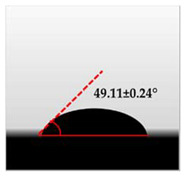	49.11 ± 0.24 ^b^
SW/4% GL-MCC	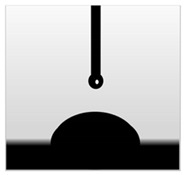	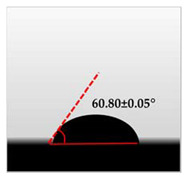	60.80 ± 0.05 ^d^
SW/6% GL-MCC	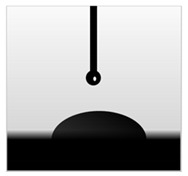	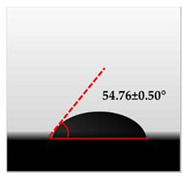	54.76 ± 0.50 ^c^
SW/8% GL-MCC	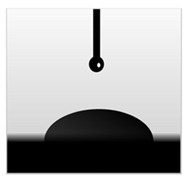	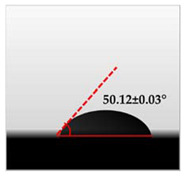	50.12 ± 0.03 ^c^

Note: Any mean values with the different superscript a, b, c and d indicates significant difference (*p* > 0.05).

## References

[1] Trache D., Hussin M.H., Chuin C.T.H., Sabar S., Fazita M.N., Taiwo O.F., Hassan T., Haafiz M.M. (2016). Microcrystalline cellulose: Isolation, characterization and bio-composites application—A review. Int. J. Biol. Macromol..

[2] Plackett D. (2011). Biopolymers: New Materials for Sustainable Films and Coatings.

[3] Bahari S.A., Krause A. (2016). Utilizing Malaysian bamboo for use in thermoplastic composites. J. Clean. Prod..

[4] Shankar S., Rhim J.-W. (2016). Preparation of nanocellulose from micro-crystalline cellulose: The effect on the performance and properties of agar-based composite films. Carbohydr. Polym..

[5] Abdul Khalil H.P.S., Yap S.W., Tye Y.Y., Tahir P.M., Rizal S., Fazita M.N. (2018). Effects of corn starch and *Kappaphycus alvarezii* seaweed blend concentration on the optical, mechanical, and water vapor barrier properties of composite films. BioResources.

[6] Wilpiszewska K., Czech Z. (2014). Citric acid modified potato starch films containing microcrystalline cellulose reinforcement—Properties and application. Starch.

[7] Zhang Q., Lei H., Cai H., Han X., Lin X., Qian M., Zhao Y., Huo E., Villota E.M., Mateo W. (2020). Improvement on the properties of microcrystalline cellulose/polylactic acid composites by using activated biochar. J. Clean. Prod..

[8] Bhasney S.M., Bhagabati P., Kumar A., Katiyar V. (2019). Morphology and crystalline characteristics of polylactic acid [PLA]/linear low density polyethylene [LLDPE]/microcrystalline cellulose [MCC] fiber composite. Compos. Sci. Technol..

[9] Wong K.M. (1995). The Bamboos of Peninsular Malaysia.

[10] Hermawan D., Lai T.K., Jafarzadeh S., Gopakumar D.A., Hasan M., Owolabi F.T., Aprilia N.S., Rizal S., Abdul Khalil H.P.S. (2019). Development of seaweed-based bamboo microcrystalline cellulose films intended for sustainable food packaging applications. BioResources.

[11] Izzati A.N.A., John W., Fazita M.N., Najieha N., Azniwati A., Abdul Khalil H.P.S. (2020). Effect of empty fruit bunches microcrystalline cellulose (MCC) on the thermal, mechanical and morphological properties of biodegradable poly (lactic acid)(PLA) and polybutylene adipate terephthalate (PBAT) composites. Mater. Res. Express.

[12] Zakaria Z., Islam M., Hassan A., Mohamad Haafiz M., Arjmandi R., Inuwa I., Hasan M. (2013). Mechanical properties and morphological characterization of PLA/chitosan/epoxidized natural rubber composites. Adv. Mater. Sci. Eng..

[13] Rizal S., Fizree H., Hossain M.S., Gopakumar D.A., Ni E.C.W., Abdul Khalil H.P.S. (2020). The role of silica-containing agro-industrial waste as reinforcement on physicochemical and thermal properties of polymer composites. Heliyon.

[14] Pawar R., Tekale S.U., Shisodia S., Totre J.T., Domb A.J. (2014). Biomedical applications of poly (lactic acid). Recent Pat. Regen. Med..

[15] Jawaid M., Abdul Khalil H.P.S. (2011). Cellulosic/synthetic fibre reinforced polymer hybrid composites: A review. Carbohydr. Polym..

[16] Pathak V.M. (2017). Review on the current status of polymer degradation: A microbial approach. Bioresour. Bioprocess..

[17] Zarina S., Ahmad I. (2015). Biodegradable composite films based on κ-carrageenan reinforced by cellulose nanocrystal from kenaf fibers. BioResources.

[18] Huq T., Salmieri S., Khan A., Khan R.A., Le Tien C., Riedl B., Fraschini C., Bouchard J., Uribe-Calderon J., Kamal M.R. (2012). Nanocrystalline cellulose (NCC) reinforced alginate based biodegradable nanocomposite film. Carbohydr. Polym..

[19] Kanmani P., Rhim J.-W. (2014). Development and characterization of carrageenan/grapefruit seed extract composite films for active packaging. Int. J. Biol. Macromol..

[20] Torres-Hernández Y.G., Ortega-Díaz G.M., Téllez-Jurado L., Castrejón-Jiménez N.S., Altamirano-Torres A., García-Pérez B.E., Balmori-Ramírez H. (2018). Biological compatibility of a polylactic acid composite reinforced with natural chitosan obtained from shrimp waste. Materials.

[21] Suvachittanont S., Ratanapan P. (2013). Optimization of micro crystalline cellulose production from corn cob for pharmaceutical industry investment. J. Chem. Chem. Eng..

[22] Pachuau L., Vanlalfakawma D.C., Tripathi S.K., Lalhlenmawia H. (2014). Muli bamboo (*Melocanna baccifera*) as a new source of microcrystalline cellulose. J. Appl. Pharm. Sci..

[23] Chuayjuljit S., Su-uthai S., Charuchinda S. (2010). Poly (vinyl chloride) film filled with microcrystalline cellulose prepared from cotton fabric waste: Properties and biodegradability study. Waste Manag. Res..

[24] Ghanbarzadeh B., Almasi H. (2011). Physical properties of edible emulsified films based on carboxymethyl cellulose and oleic acid. Int. J. Biol. Macromol..

[25] Tan Z., Yi Y., Wang H., Zhou W., Yang Y., Wang C. (2016). Physical and degradable properties of mulching films prepared from natural fibers and biodegradable polymers. Appl. Sci..

[26] Uthaya Kumar U.S., Abdulmadjid S., Olaiya N., Amirul A., Rizal S., Rahman A., Alfatah T., Mistar E., Abdul Khalil H.P.S. (2020). Extracted Compounds from Neem Leaves as Antimicrobial Agent on the Physico-Chemical Properties of Seaweed-Based Biopolymer Films. Polymers.

[27] Seligra P.G., Jaramillo C.M., Famá L., Goyanes S. (2016). Biodegradable and non-retrogradable eco-films based on starch–glycerol with citric acid as crosslinking agent. Carbohydr. Polym..

[28] Achor M., Oyeniyi Y., Yahaya A. (2014). Extraction and characterization of microcrystalline cellulose obtained from the back of the fruit of *Lageriana siceraria* (water gourd). J. Appl. Pharm. Sci..

[29] Ohwoavworhua F., Adelakun T. (2005). Some physical characteristics of microcrystalline cellulose obtained from raw cotton of *Cochlospermum planchonii*. Trop. J. Pharm. Res..

[30] Zuo Y., Gu J., Cao J., Wei S., Tan H., Zhang Y. (2015). Effect of starch/polylactic acid ratio on the interdependence of two-phase and the properties of composites. J. Wuhan Univ. of Technol. Mater. Sci. Ed..

[31] Reddy J.P., Rhim J.-W. (2014). Characterization of bionanocomposite films prepared with agar and paper-mulberry pulp nanocellulose. Carbohydr. Polym..

[32] Chen W., Yu H., Liu Y., Hai Y., Zhang M., Chen P. (2011). Isolation and characterization of cellulose nanofibers from four plant cellulose fibers using a chemical-ultrasonic process. Cellulose.

[33] Jumaidin R., Sapuan S., Jawaid M., Ishak M., Sahari J. (2017). Characteristics of *Eucheuma cottonii* waste from East Malaysia: Physical, thermal and chemical composition. Eur. J. Phycol..

[34] Whatley B.R., Wen X. (2012). Intervertebral disc (IVD): Structure, degeneration, repair and regeneration. Mater. Sci. Eng. C.

[35] dos Santos F.A., Iulianelli G.C., Tavares M.I. (2017). Effect of microcrystalline and nanocrystals cellulose fillers in materials based on PLA matrix. Polym. Test..

[36] Harnkarnsujarit N., Li Y. (2017). Structure–property modification of microcrystalline cellulose film using agar and propylene glycol alginate. J. Appl. Polym. Sci..

[37] Deepa B., Abraham E., Pothan L.A., Cordeiro N., Faria M., Thomas S. (2016). Biodegradable nanocomposite films based on sodium alginate and cellulose nanofibrils. Materials.

